# Dihydromyricetin as a Functional Additive to Enhance Antioxidant Capacity and Inhibit the Formation of Thermally Induced Food Toxicants in a Cookie Model

**DOI:** 10.3390/molecules23092184

**Published:** 2018-08-30

**Authors:** Jing Teng, Xuduo Liu, Xiaoqian Hu, Yueliang Zhao, Ning-Ping Tao, Mingfu Wang

**Affiliations:** 1 College of Food Science and Technology, Shanghai Ocean University, Shanghai 201306, China; victory2014t@163.com (J.T.); ryukyota@163.com (X.L.); xqhu@shou.edu.cn (X.H.); ylzhao@shou.edu.cn (Y.Z.); nptao@shou.edu.cn (N.-P.T.); 2 Shanghai Engineering Research Center of Aquatic-Product Processing & Preservation, Shanghai 201306, China

**Keywords:** dihydromyricetin, naringenin, naringin, hesperetin, cookie model, antioxidant capacity, toxicants

## Abstract

Recently, there is a growing interest in fortifying food products with flavonoids to enhance health benefits. Naringenin, naringin, hesperetin, and dihydromyricetin are four typical flavonoids constituting a natural part of our diet. In the present work, they were fortified into a chia oil cookie model to evaluate their thermal stability during baking as well as their impact on antioxidant capacity and toxicant formation. Among them dihydromyricetin was the most unstable one (only 36.1% of which was left after baking at 180 °C for 20 min) and led to a loss of brightness in cookie. However, the antioxidant capacity of cookie fortified with dihydromyricetin was significantly enhanced compared with untreated cookie; on the other hand, dihydromyricetin showed the strongest effect to attenuate lipid and protein oxidation as well as decrease the level of fluorescent advanced glycation endproducts and carboxymethyl lysine in cookie model. Overall, among the four selected flavonoids, dihydromyricetin might be the most promising functional bakery additive enhancing the antioxidant capacity while decreasing the formation of toxicants.

## 1. Introduction

Nowadays, the incorporation of edible plant extracts into food systems is a common practice in various cuisines all over the world because of their health benefits and low side effects [1]. Polyphenolic compounds, the main components in plant extracts, are considered as one group of most powerful antioxidants in human diets with flavonoids accounting for the major part of the total phenolics’ intake. Based on the structural characteristics, flavonoids can be divided into different subgroups including anthocyanins, flavanols, flavanones, flavones, flavonols, and isoflavones. Recently, due to their possible contribution to reduce the risk for many chronic diseases [2], flavonoids have become popular ingredients to be formulated into nutraceutical and pharmaceutical products, as well as foods to enhance product health functions [3].

As thermal processing is a key operation in the food industry, the flavonoids fortified in food might undergo a series of chemical changes with increased temperature, thus impacting the final qualities of the food system and the biological activities as well as thermal stabilities of flavonoids; meanwhile the Maillard reaction and lipid peroxidation occurring during thermal treatment lead to the formation of some unpleasant toxic substances, such as advanced glycation endproducts (AGEs), malonaldehyde (MDA), and so on, while the formation of these toxic compounds could be inhibited by the addition of flavonoids. Thus these aspects need to be carefully addressed. Cookie is prevalent around the world, and is a typical kind of bakery product containing high ratios of edible oil and sugar, baked under high temperature, so it can be a good model to study the interaction of flavonoids with food properties.

**Figure 1:**
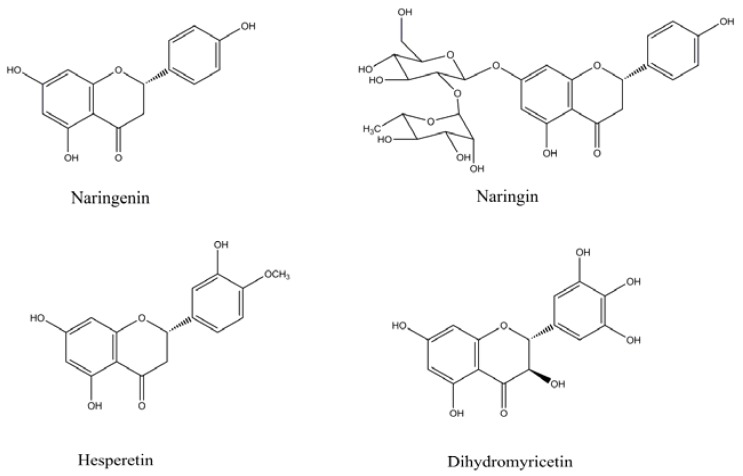
Chemical structures of naringenin, naringin, hesperetin, and dihydromyricetin.

Naringenin (4′,5,7-trihydroxyflavanone) is one of the most abundant citrus flavanones with a wide range of biological activities [4,5]. In our previous work, we found that the fortification of bread with it could significantly inhibit the formation of advanced glycation endproducts in breadcrust, while enhancing the antioxidant and antibacterial activity of bread crumb without bringing undesirable changes to the bread quality attributes [6]. However, it is still unknown whether naringenin and its structural derivatives could have a positive effect in cookie which contains much higher content of edible oil and sugar and are produced under higher temperature than bread. Naringin, hesperetin and dihydromyricetin (Figure 1), with different substituted groups, are also three typical flavonoids constituting a natural part of our diet [3]. Naringin (naringenin-7-rhamnoglucoside), as the major glycoside of naringenin, is a predominant flavonoid in grapefruit with various positive therapeutic properties and is reported to be a popular nutraceutical ingredient for the management of diabetes and its complications [7,8]. Hesperitin, which has one more methoxy group than naringenin, is another predominant flavonoid in citrus fruits, and has been reported to possess many beneficial effects [9]. In fact, hesperetin and naringin extract have already been approved to serve as food additives in China according to GB 2760. Moreover, dihydromyricetin is known as a main component in the dried stems and leaves of *Ampelopsis grossedentata* which have been consumed as a health tea and herbal medicine for hundreds of years, and recently approved as new food raw materials in China [1]. Dihydromyricetin has more hydroxyl substitutions than naringenin and was also found to have various health benefits such as reducing hypertension, removing sputum, protecting the liver, and antioxidative and antibacterial properties [10]. In fried beef patties, Zhou et al. found that dihydromyricetin could significantly inhibit the formation of some key heterocyclic amines including PhIP, MeIQx, and 4,8-DiMeIQx [1]. In other words, fortification with the flavonoids mentioned above in food systems is reasonable and possible. However, to the best of our knowledge, there is a lack of information about the comparison of the thermal stability of naringenin, naringin, hesperetin, and dihydromyricetin as well as their impact on antioxidant capacity and toxicant formation in cookies.

Therefore, the objective of this study is to obtain a better understanding of the impact of naringenin (NN), naringin (NG), hesperetin (HT), and dihydromyricetin (DT) on a typical bakery model, cookie with high edible oil and sugar content processed at high baking temperature. Their thermal stabilities after baking were firstly studied. Then their effects to inhibit the formation of AGEs and malonaldehyde, as well as their impact on antioxidant activity, color, and texture of cookies were also investigated.

## 2. Results and Discussion

### 2.1. Stability of NN, NG, HT, and DT during Baking Process of Cookie Model

**Figure 2:**
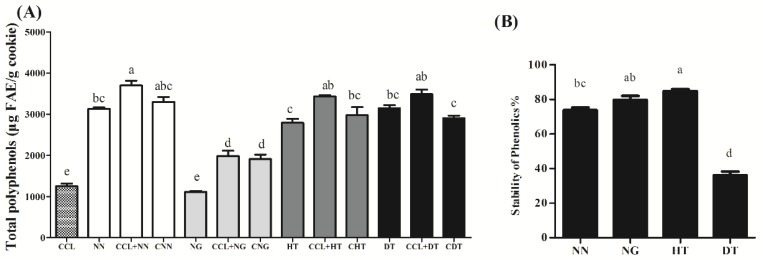
Total polyphenol content (**A**) and thermal stability of NN, NG, HT, and DT in cookies (**B**) after baking at 180 °C for 20 min. NN, NG, HT, and DT indicated naringenin, naringin, hesperetin, and dihydromyricetin respectively; CCL, CNN, CNG, CHT, and CDT indicated control chia oil cookie, cookie fortified with naringenin, naringin, hesperetin, and dihydromyricetin respectively. “CCL + polyphenol” indicated control cookie mixed with equivalent amounts of original flavonoids. The significant difference results were indicated in lowercase letters as shown in Figure 2A,B. Data not contained the same letter demonstrated the difference was significant (*p* < 0.05), while the same letter included indicated that the difference was not significant (*p* > 0.05).

The total polyphenol contents (TPC) of cookie samples were summarized in Figure 2A. TPC (±SD) varied significantly among different cookie samples, and cookies fortified with NG (CNG) had significantly lower content of total polyphenol than those fortified with NN, HT, and DT (CNN, CHT, and CDT), which might own to the fact that naringin, a flavonoid glycoside, has a larger molecular weight and consequently low TPC at the same substitution level (0.25 wt%). The TPC in CDT was significantly lower than that in CCL + DT, which indicated that, compared with cookie fortified with naringenin, naringin, and hesperetin, baking had a stronger influence on the total polyphenol content in the cookie sample fortified with dihydromyricetin (*p* < 0.05). A previous report showed that the losses of phenolic compounds by thermal decomposition or volatilization were the main causes of a lower phenol content after exposing to high temperature [11], or they might actively participate in the chemical reactions that occurred during the thermal treatment of food. This might be what happened to dihydromyricetin.

The stability of polyphenols is an important consideration, for on the one hand, many flavonoids are susceptible to thermal degradation [1] and on the other hand, the precise information of polyphenols’ stability is closely related to TPC in cookie samples as well as their biological functions. The stabilities of naringenin, naringin, hesperitin, and dihydromyricetin were determined by HPLC-DAD according to their standard curves. As shown in Figure 2B, hesperetin, naringin, and naringenin were relatively stable during thermal treatment, and remained at 84.8, 79.5, and 73.5%, respectively, after baking. However, dihydromyricetin was quite unstable, as supported by the HPLC–diode array detector analysis, only 36.1% of which was left in cookies after baking at 180 °C for 20 min. The results from HPLC were consistent with the above results of total phenol content. These data further supported the previous finding that dihydromyricetin was easy to degrade or transform during thermal treatment, as nearly all DT was degraded or transformed after heat treatment at 128 °C for 2 h in PBS [1].

### 2.2. ABTS^+^ and DPPH Radical Scavenging Activity

**Figure 3:**
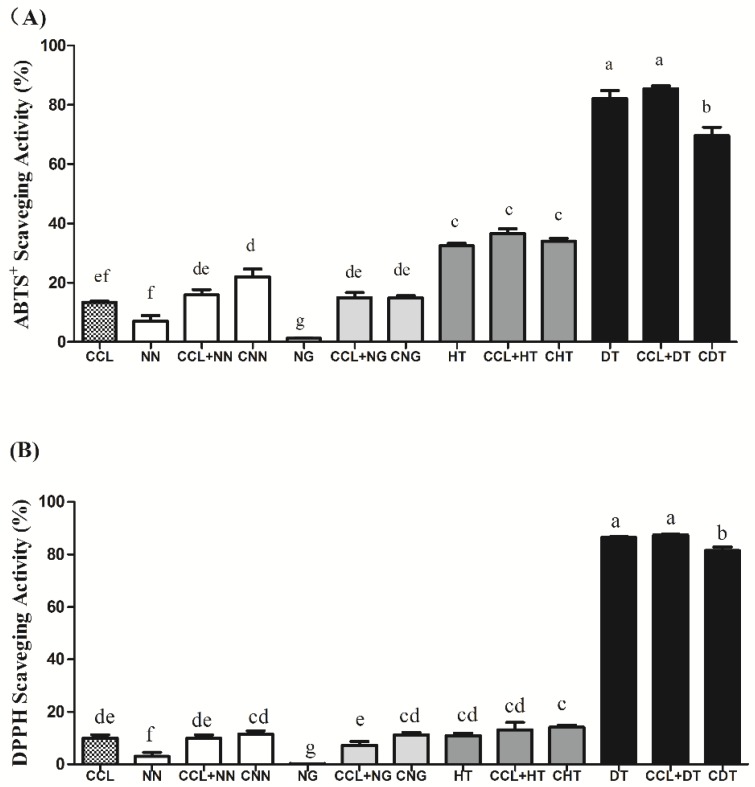
ABTS^+^ (**A**) and DPPH (**B**) scavenging activity of the extracts from cookies samples with or without the addition of various flavonoids. NN, NG, HT, and DT indicated naringenin, naringin, hesperetin, and dihydromyricetin respectively; CCL, CNN, CNG, CHT, and CDT indicated control chia oil cookie, cookie fortified with naringenin, naringin, hesperetin, and dihydromyricetin respectively. “CCL + polyphenol” indicated control cookie mixed with equivalent amounts of original flavonoids. The significant difference results were indicated in lowercase letters as shown in Figure 3A,B. Data not contained the same letter demonstrated the difference was significant (*p* < 0.05), while the same letter included indicated that the difference was not significant (*p* > 0.05).

The antioxidant capacities of control cookies, added flavonoids alone, control cookies mixed with equivalent amount of added flavonoids, and flavonoid-fortified cookies were examined with both ABTS^+^ and DPPH radical scavenging methods (Figure 3). To start with, control cookie (CCL) could exhibit antioxidant capacity, as its original ingredients and Maillard reaction products (MRPs) formed in baking process had antioxidant properties (Figure 3). In addition, among the four tested flavonoid compounds (NN, NG, HT, and DT), DT showed the highest ABTS^+^ scavenging ability of 82.07%, followed by HT 32.42% and NN 7.06%. However, the effect of NG was poor and showed the lowest ABTS^+^ scavenging ability of 1.29% (Figure 3A). The same trend could be observed in DPPH scavenging capacity (Figure 3B), which was DT (86.43%) > HT (10.95%) > NN (2.99%) > NG (0.16%). NG exhibited the lowest antioxidant capacity in both tests, as flavonoid glycosides did not exhibit strong antioxidant effects [12]. Moreover, the antioxidative action of polyphenols is commonly attributed to the presence of OH groups, in particular, the existence of a second OH group in the ortho or para position contributes to better antioxidative efficiencies [12,13], which explains the higher antioxidative activity of DT [1] than other three kinds of flavanones in our test models.

In this work, we found that cookies fortified with dihydromyricetin (CDT) and hesperetin (CHT) showed a significant increase in both ABTS^+^/DPPH scavenging activity compared with the control cookie (CCL). Naringenin fortification in a chia oil cookie significantly increased the ABTS^+^ radical scavenging activity of cookie samples, yet the DPPH radical scavenging activity of cookie could not be significantly increased. However, there seems no effect on antioxidant enhancement in cookie when fortified with naringin. In a word, cookies fortified with dihydromyricetin (CDT) had the best antioxidant capability among all the cookie samples as shown in both ABTS^+^/DPPH scavenging tests (followed by CHT, CNN, and CNG), yet the increase in the antioxidant activity was lower than that carried by the same quantity of DT originally added to the cookie recipe (denoted CCL + DT > CDT in Figure 3A,B). This observation was consistent with phenolic stability mentioned in Section 2.1 that dihydromyricetin was relatively sensitive to thermal processing, thus baking would decrease the antioxidant capacity of dihydromyricetin in cookies.

### 2.3. Impact of Selected Flavonoids on Toxicant Formation in Cookie Model

In our pre-experiments, we prepared two groups of cookie samples, one was baked with refined palm oil only (PCL), and the other one was baked with the mixed oil of chia oil and refined palm oil (CCL). We found that the contents of malondialdehyde (MDA) and fluorescent advanced glycation endproducts (AGEs) in chia oil cookie (CCL) were higher than those in palm oil cookie (PCL), which might owe to the fact that chia oil was an abundant source of polyunsaturated fatty acids (especially n-3 PUFAs) compared with palm oil. Therefore, in the subsequent study, we only used chia oil cookie as a model to test whether the fortification of flavonoids could lead to a reduction of formation of these toxicants.

**Figure 4:**
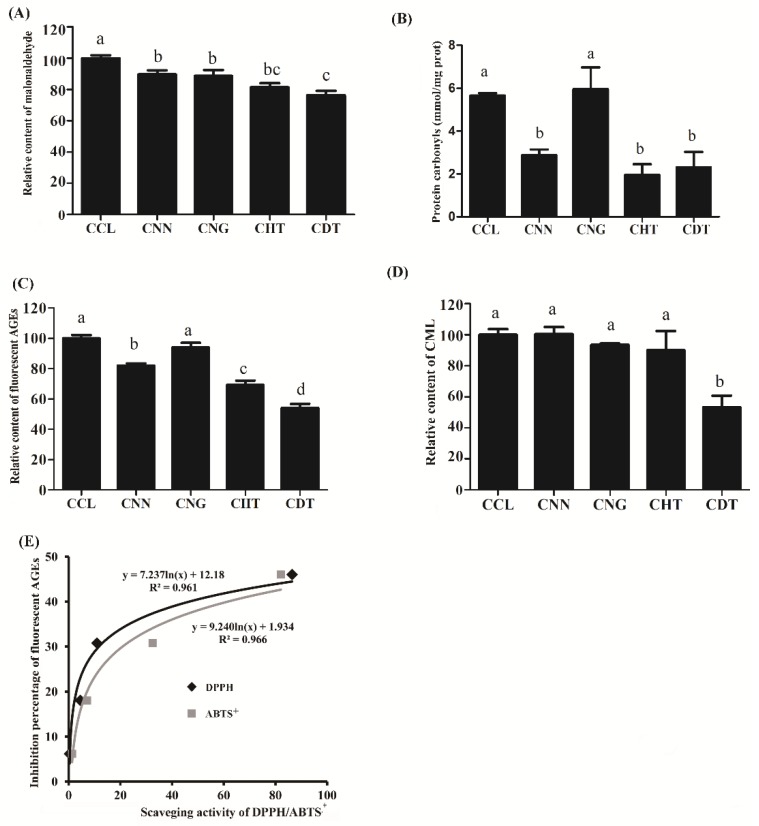
Inhibitory effects of different flavonoid additions (0.25% *w*/*w*) on the formation of malonaldehyde (**A**), protein carbonyls (**B**), fluorescent advanced glycation endproducts (**C**), and carboxymethyl lysine (**D**) in cookie sample, and the correlation rate between antioxidant capacity and inhibition percentage of fluorescent advanced glycation endproducts by flavanones-fortified cookies (**E**). CCL, CNN, CNG, CHT, and CDT indicated control chia oil cookie, cookie fortified with naringenin, naringin, hesperetin, and dihydromyricetin respectively. AGEs stood for advanced glycation endproducts and CML stood for carboxymethyl lysine. The significant difference results were indicated in lowercase letters as shown in Figure 4A–D. Data not contained the same letter demonstrated the difference was significant (*p* < 0.05), while the same letter included indicated that the difference was not significant (*p* > 0.05).

MDA is widely used as a lipid peroxidation indicator in foods [14]. During lipid peroxidation, polyunsaturated fatty acids yield MDA, which is directly or indirectly involved in the multistage process of carcinogenesis and leads to mutations of tumor suppressor genes [15]. Figure 4A showed that the fortification with flavonoids (NN, NG, HT, and DT) can significantly lowered the MDA content in chia oil cookies, and the average inhibition rates were 10.3, 11.2, 18.6, and 23.6%, respectively. Moreover, the flavonoid-treated cookies showed higher retention rates of α-linolenic acid than control cookie CCL (data not shown). Since lipid oxidation would in turn promote the oxidation of protein and formation of extra carbonyl groups [16], the protein carbonyl contents in cookies fortified with or without flavonoids were presented in Figure 4B, and the results suggested a protective role of NN, HT, and DT against protein oxidation during baking process.

The formation of advanced glycation endproducts was another concern in cookies, as dietary AGEs were harmful to human health to accelerate many diseases, including retinopathy, cataracts, and Alzheimer’s disease, therefore it was of vital significance to reduce AGEs in foods [17]. Our results suggested that under baking conditions, NN, HT, and DT could effectively reduce the amount of fluorescent AGEs in chia oil cookies (Figure 4C). Among them, DT was the most effective one against fluorescent AGEs formation (around 47.0% inhibition), followed by HT (31.4%), and NN (18.3%), while NG showed no significant inhibition. We also found that the inhibitory activity against the formation of fluorescent AGEs was positively correlated with polyphenols’ antioxidant activity (both ABTS^+^ and DPPH) as shown in Figure 4E, which indicated that their inhibition capability towards fluorescent AGEs might be attributable to the phenolics’ free radical scavenging capability. Apart from fluorescent AGEs, N^ε^-(carboxymethyl)lysine (CML) was also commonly used as a marker of AGEs formation in foods [18,19]. Results indicated that CML formation was significantly inhibited in DT fortified cookies (Figure 4D). Yet a significant difference was not found on the formation of CML among the cookie samples fortified with the other three flavonoids compared with CCL.

### 2.4. Quality Attributes for Cookies Fortified with Selected Flavonoids

**Figure 5:**
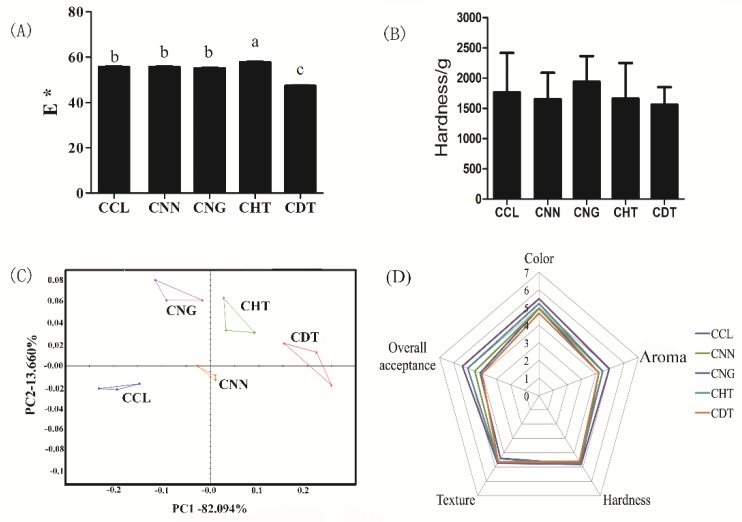
Impact of naringenin, naringin, hesperetin, and dihydromyricetin on selected cookie quality attributes. (**A**) Color difference between flavonoid-fortified and control cookies; (**B**) texture of cookies; (**C**) PCA chart of odors derived from different cookie models; and (**D**) average of the parameters on sensory evaluation of control and flavonoid substitution cookies. CCL, CNN, CNG, CHT, and CDT indicated control chia oil cookie, cookie fortified with naringenin, naringin, hesperetin, and dihydromyricetin, respectively. The significant difference results were indicated in lowercase letters as shown in Figure 5A,B. Data not contained the same letter demonstrated the difference was significant (*p* < 0.05), while the same letter included indicated that the difference was not significant (*p* > 0.05).

Though some selected flavonoids have been proved to show good effect to enhance antioxidant capacity and inhibit the formation of toxicants when fortified in cookies, their impacts on food quality should also be evaluated to check their acceptability as functional food ingredients. In general, as additives, polyphenols could influence the color of cookies, and the impact changed with different phenolic types [17]. The impact of four selected flavonoids on cookie color was summarized in Figure 5A. Results showed that the addition of HT increased the L* (lightness), a* (redness), and b* (yellowness) of the cookie sample, however, cookie fortified with DT had opposite trends. For a comprehensive analysis, cookie fortified with DT had relatively lower E value than the control cookie, which indicated a loss in brightness caused by DT. The loss in brightness led to a slight reduction in the color score of CDT on sensory evaluation as shown in Figure 5D. Textural analysis (Figure 5B) showed that appropriate addition levels of selected polyphenols (0.25% *w*/*w*) would not cause undesirable change in hardness of cookie. Besides, the PCA chart of odors derived from five groups of cookie samples was also summarized in Figure 5C, and results showed that the control and treated cookies could be well distinguished from each other with a discrimination index of 83, indicating that the addition of selected flavonoids can affect the volatile component profile of the cookies. The sensory evaluation indicated that all formulations were acceptable as they received scores greater than 4 (Figure 5D).

## 3. Materials and Methods

### 3.1. Materials

Naringenin, naringin, hesperetin, and dihydromyricetin (≥98%) were purchased from Xi’an Natural Field Biological Technology Co. Ltd. (Xi’an, China). 2,2′-azino-bis(3-ethylbenzthiazo-line-6-sulphonic acid) and 2,2-diphenyl-1-picrylhydrazyl were obtained from Sigma-Aldrich Company (St. Louis, MO, USA). The ingredients for cookie-making were purchased from a local supermarket in Shanghai, China. 1,1,3,3-Tetraethoxypropane and polyphenol standards were bought from ANPEL Scientific Instrument Co. (Shanghai, China). Other chemicals including anhydrous sodium carbonate, ethylene diamine tetraacetic acid, Folin-Ciocalteu’s phenol reagent, potassium peroxodisulphate, thiobarbituric acid, and trichloroacetic acid were of analytical grade and obtained from Sinopharm Chemical Reagent Co. (Shanghai, China).

### 3.2. Preparation of Cookie Model

The cookie model was built according to the published literature with slight modification [20]. Generally, the cookie dough (220 g in total) was composed of low-gluten wheat flour (120 g), sucrose (60 g), chia oil (20 g), and refined palm oil (20 g). Sucrose and 0.25% (*w*/*w*) of individual flavonoids (replacing an equivalent amount of low-gluten wheat flour in the cookie formula) including naringenin (NN), naringin (NG), hesperetin (HT), and dihydromyricetin (DT), were initially mixed in oil and then mixed with wheat flour to prepare the dough for experimental group. In our study, we designed five groups of cookies; they were CCL (no flavonoid fortification, control cookie), CNN (cookie fortified with naringenin), CNG (cookie fortified with naringin), CHT (cookie fortified with hesperitin), and CDT (cookie fortified with dihydromyricetin). The cookie dough was wrapped with cling film and rolled to form a 12 cm (width) × 24 cm (long) cake. Then a grinder was used to make 8 round cookies (5.5 cm in diameter and 0.5 cm in thickness) for each 220 g of dough. They were then baked in a preheated baking oven at 180 °C for 20 min. After baking, the cookie samples were cooled to room temperature, smashed and kept at −20 °C until further analysis. The following tests were carried out by at least three replicates for two batches of cookie samples.

### 3.3. Stability of Selected Flavonoids during Cookie Baking Proces

The stability of selected flavonoids in cookie samples through thermal processing was determined according to Zhang et al. with slight modification [21]. One gram of cookie powders was extracted by 10 mL of 70% methanol, respectively, under strongly vortex for 6 min and sonication for 30 min. After centrifugation, the supernatant was conducted two-fold dilution with methanol, then filtered and analyzed by HPLC-DAD quantified using standard curves. The HPLC (Agilent) system was equipped with a C_18_ column (Inertsil ODS-3, 5 μm, 4.6 mm × 250 mm) while the sample injection volume, column temperature and flow rate were 10 μL, 30 °C, 1 mL/min, respectively. The gradient system was conducted as follows: 0 min, 90% A (0.1% phosphoric acid in ultrapure water)–10% B (acetonitrile); 30 min, 10% A–90% B; 31 min, 90% A–10% B; 45 min, 90% A–10% B. The detection wavelength for NN, NG, HT, and DT were 288, 284, 288, and 290 nm, respectively.

### 3.4. Determination of Total Polyphenol Content (TPC)

Total polyphenol content was determined using Folin–Ciocalteu’s phenol reagent [22]. Specifically, 187 μL of supernatant obtained was mixed with 813 μL of ethanol to form 1 mL sample dilution, added to 5 mL of freshly prepared Folin–Ciocalteu reagent (FCR) (1:10 *v*/*v* with water). The mixture was equilibrated for 6 min and then mixed with 4 mL of 7.5% sodium carbonate solution. After incubation at room temperature for 60 min, the absorbance of the mixture was read at 765 nm. Calibration was achieved with an aqueous gallic acid standard solution (10–50 μg/mL). The results were expressed as μg of ferulic acid equivalents (FAE) per g of cookie sample. All samples were determined in triplicate.

### 3.5. Antioxidant Capacity of Cookie Models

The antioxidant capacity of cookie samples was carried out by vortex-dispersing cookie powders into 70% ethanol with a final concentration of 100 mg/mL [23]. Samples were extracted by sonication for 30 min and then centrifuged to get the supernatants. 12 μL of extracted solution was added to 1188 μL of diluted ABTS cation solution while 42 μL of extracted supernatant was added to 1158μL of freshly prepared DPPH solution. After 6 min or 30 min of incubation, the absorbance was determined on a UV-2300 ultraviolet spectrophotometer. The scavenging activity (%) = [1 − (A_s_/A_0_)]/100, where A_0_ was the absorbance at 734 nm (for ABTS^+^) or 519 nm (for DPPH) of the blank and A_s_ demonstrated the absorbance of the tested sample [24].

### 3.6. Measurement of Lipid Oxidation and Protein Carbonyl Content

The measurement of malonaldehyde was carried out according to GB 5009.181-2016. Trichloroacetic acid mixture was prepared by accurately weighing and dissolving 37.5 g trichloroacetic acid and 0.5 g EDTA in distilled water to make 500 mL in total. The smashed cookie samples (2 g) were homogenized with 20 mL trichloroacetic acid mixture and extracted on a constant temperature oscillator for 30 min (50 °C). After centrifugation and filtration, the filtrate was added with equal volume of thiobarbituric acid (0.02 M) in a test tube. The tubes were heated at 90 °C in a water bath for 30 min. After being cooled down, the absorbance was measured using an UV-2300 ultraviolet spectrophotometer at 532 nm. Replicates were carried out by 3 times for two batches of cookie samples.

The analysis of fatty acid composition of baked cookies was carried out by total lipids extraction and fatty acid methyl esterification. Conditions of gas chromatograph (THERMO TRACE GC ULTRA, Thermo Fisher Scientific, Waltham, MA, USA) with FID detector (Agilent Technologies Co., Ltd., California, CA, USA.) were listed as follows: Agilent SP-2560 chromatographic column (100 m × 0.25 mm i.d × 0.2 µm) was used and the initial column temperature was 70 °C. The heating procedures were: 70–140 °C at 50 °C/min, 140 °C for 1 min; 140–180 °C at 4 °C/min, 180 °C for 1 min; 180–225 °C at 3 °C/min, 225 °C for 30 min. The temperature of gasification chamber was set as 250 °C and flow rate of N_2_ was 1mL/min; the split ratio was 45:1 and injection volume was 1 μL.

The protein carbonyl contents were measured following the instruction of protein carbonylation kit (Nanjing Jiancheng Bioengineering Institute, Nanjing, China). The protein carbonyl content was expressed as nmol carbonyl/mg protein and was calculated as (A_S_ − A_0_) × 125 × 10^5^/(22 × 0.5 × C), where As and A_0_ represented the absorbance of the experimental and blank groups, 0.5 referred to colorimetric tube diameter while c stood for protein concentration (μg/mL).

### 3.7. Determination of Advanced Glycation Endproducts in Cookie Samples

To determine the fluorescent AGEs content in cookie sample, 9.5 mL of extraction buffer (0.05% Tween 20, 1% SDS, 5% β-mercaptoethanol and 50 mM Tris-HCl at pH 7.4) was used to extract protein from 0.5 g of cookie powder [21] for 12 h in a shaker. After extraction and centrifugation, the supernatant containing extracted protein was collected and detected with excitation wavelength of 360 nm and emission wavelength of 460 nm using a microplate reader [17]. Carboxymethyllysine ELISA kit (double antibody sandwich-enzyme linked immunosorbent assay), purchased from Nanjing SenBeiJia Biological Technology Co., Ltd. (Nanjing, China) was employed to determine the N^ε^-(carboxymethyl)lysine (CML) and performed according to the manufacturer’s instructions [25].

### 3.8. Determination of Selected Quality Attributes of Cookie Samples

Color changes of cookie with or without the addition of different concentrations of flavonoids were measured with a colorimeter (CR-400, Konica Minolta, Japan), and E index was calculated as (L*^2^ + a*^2^ + b*^2^)^1/2^. The coordinate L* suggests the position between black (0) and white (100), a* indicates the position between red (+) and green (−) while b* shows the position between yellow (+) and blue (−). The cookie texture was evaluated using a TA-XTPlus Texture Analyzer (Lotun Science Co., Ltd., Beijing, China) which was installed with a 5 kg load cell and a cylinder probe of 2 mm in diameter referred to the procedure published by Ou et al. [17]. One gram of cookie powder was weighted in 10 mL head-space vial and the parameters of electric nose (Fox 4000 Sensory Array Fingerprint Analyzer (Alpha MOS, Toulouse, France) were: flow rate of carrier gas-clean dry air 150 mL/min; headspace generation time 220 s, temperature 68 °C, agitation speed 500 r/min; headspace injection volume 2500 μL, injection speed 2500 μL/s, injection needle temperature 78 °C; acquisition time 120 s, lag time 600 s. The sensory evaluation of cookie samples was conducted by a panel of 10 judges using the seven-point hedonic scale, and cookies were considered acceptable if their average scores for the overall acceptance were greater than 4 [26].

### 3.9. Statistical Analyses

The results obtained from the present study were represented as the mean values ± standard deviation (S.D.). Statistical analyses were performed using one-way ANOVA by Prism 5.0, GraphPad Software (San Diego, CA, USA). *p* < 0.05 was considered to be statistically significant.

## 4. Conclusions

This study demonstrated that cookies fortified with naringenin, naringin, hesperetin, and dihydromyricetin had various properties compared with control cookies. They might change the color, yet were acceptable according to sensory evaluation. After baking process (180 °C for 20 min), the antioxidant capacity of cookies fortified with hesperetin and dihydromyricetin were significantly enhanced compared with untreated cookies. The fortification of selected flavonoids in cookies could effectively inhibit the formation of malonaldehyde, yet their effect on other toxicant formations varied. Compared with other three tested flavanones at the same mass-based fortification level, though dihydromyricetin was the most unstable one, it indeed showed the strongest effect attenuating lipid and protein oxidation, as well as decreasing the level of carboxymethyllysine and fluorescent advanced glycation endproducts in cookie model. Overall, dihydromyricetin might be the most promising functional cookie additive suggested by its significant promotion of antioxidant capacity together with strong inhibition against toxicant formation.

## References

[1] Zhou B., Zhao Y., Wang X., Fan D., Cheng K., Wang M. (2018). Unraveling the inhibitory effect of dihydromyricetin on heterocyclic aromatic amines formation. J. Sci. Food. Agric..

[2] Alipour B., Rashidkhani B., Edalati S. (2015). Dietary flavonoid intake, total antioxidant capacity and lipid oxidative damage: A cross-sectional study of Iranian women. Nutrition.

[3] Liu B., Li W., Nguyen T.A., Zhao J. (2012). Empirical, thermodynamic and quantum-chemical investigations of inclusion complexation between flavanones and (2-hydroxypropyl)-cyclodextrins. Food Chem..

[4] Jabbari M., Jabbari A. (2016). Antioxidant potential and DPPH radical scavenging kinetics of water-insoluble flavonoid naringenin in aqueous solution of micelles. Colloids Surf. A.

[5] Karim N., Jia Z., Zheng X., Cui S., Chen W. (2018). A recent review of citrus flavanone naringenin on metabolic diseases and its potential sources for high yield-production. Trends Food Sci. Technol..

[6] Teng J., Li Y., Yu W., Zhao Y., Hu X., Tao N.-P., Wang M. (2018). Naringenin, a common flavanone, inhibits the formation of AGEs in bread and attenuates AGEs-induced oxidative stress and inflammation in RAW264.7 cells. Food Chem..

[7] Wang J., Qi Y., Niu X., Tang H., Meydani S.N., Wu D. (2017). Dietary naringenin supplementation attenuates experimental autoimmune encephalomyelitis by modulating autoimmune inflammatory responses in mice. J. Nutr. Biochem..

[8] Rotimi S.O., Adelani I.B., Bankole G.E., Rotimi O.A. (2018). Naringin enhances reverse cholesterol transport in high fat/low streptozocin induced diabetic rats. Biomed. Pharmacot..

[9] Usach I., Taléns-Visconti R., Magraner-Pardo L., Peris J.E. (2015). Hesperetin induces melanin production in adult human epidermal melanocytes. Food Chem. Toxicol..

[10] Xin M., Ma Y., Lin W., Xu K., Chen M. (2014). Study on the structure–activity of dihydromyricetin and its new production. J. Therm. Anal. Calorim..

[11] Soong Y.Y., Barlow P.J. (2004). Antioxidant activity and phenolic content of selected fruit seeds. Food Chem..

[12] Zhang Y.Y., Zhang F., Thakur K., Ci A.T., Wang H., Zhang J.G., Wei Z.J. (2018). Effect of natural polyphenol on the oxidative stability of pecan oil. Food Chem. Toxicol..

[13] Damasceno S.S., Santos N.A., Santos I.M.G., Souza A.L., Souza A.G., Queiroz N. (2013). Caffeic and ferulic acids: An investigation of the effect of antioxidants on the stability of soybean biodiesel during storage. Fuel.

[14] Jung S., Nam K.C., Jo C. (2016). Detection of malondialdehyde in processed meat products without interference from the ingredients. Food Chem..

[15] Sakai T., Kawahara S. (2005). Malonaldehyde content in some breads and its change during storage. J. Food Hyg. Soc. Jpn..

[16] Masahiro T., Risa M., Harutaka Y., Kazuhiro C. (1996). Novel antioxidants isolated from *Perilla frutescens* Britton var. crispa (Thunb.). Biosci. Biotechnol. Biochem..

[17] Ou J., Teng J., El-Nezami H.S., Wang M. (2018). Impact of resveratrol, epicatechin and rosmarinic acid on fluorescent AGEs and cytotoxicity of cookies. J. Funct. Foods.

[18] Hull G.L., Woodside J.V., Ames J.M., Cuskelly G.J. (2012). N^ε^-(carboxymethyl) lysine content of foods commonly consumed in a Western style diet. Food Chem..

[19] Yu P., Xu X.B., Yu S.J. (2017). Inhibitory effect of sugarcane molasses extract on the formation of N^ε^-(carboxymethyl) lysine and N^ε^-(carboxyethyl) lysine. Food Chem..

[20] Charissou A., Ait-Ameur L., Birlouez-Aragon I. (2007). Kinetics of formation of three indicators of the Maillard reaction in model cookies: Influence of baking temperature and type of sugar. J. Agric. Food Chem..

[21] Zhang X., Chen F., Wang M. (2014). Antioxidant and antiglycation activity of selected dietary polyphenols in a cookie model. J. Agric. Food Chem..

[22] Padhi E.M., Liu R., Hernandez M., Tsao R., Ramdath D.D. (2017). Total polyphenol content, carotenoid, tocopherol and fatty acid composition of commonly consumed Canadian pulses and their contribution to antioxidant activity. J. Funct. Foods.

[23] Peng X., Ma J., Cheng K.W., Jiang Y., Chen F., Wang M. (2010). The effects of grape seed extract fortification on the antioxidant activity and quality attributes of bread. Food Chem..

[24] Zhu F., Sakulnak R., Wang S. (2016). Effect of black tea on antioxidant, textural, and sensory properties of Chinese steamed bread. Food Chem..

[25] Martinez-Saez N., García A.T., Pérez I.D., Rebollo-Hernanz M., Mesías M., Morales F.J., del Castillo M.D. (2017). Use of spent coffee grounds as food ingredient in bakery products. Food Chem..

[26] Ho L.H., Aziz N.A.A., Azahari B. (2013). Physico-chemical characteristics and sensory evaluation of wheat bread partially substituted with banana (*Musa acuminata* X balbisiana cv. Awak) pseudo-stem flour. Food Chem..

